# Alemtuzumab-BEAM as conditioning for allogeneic hematopoietic stem cell transplantation in relapsed/refractory Hodgkin lymphoma: a single-center analysis

**DOI:** 10.1007/s00432-016-2134-3

**Published:** 2016-02-26

**Authors:** W. Rabitsch, M. Bojic, P. Wohlfarth, M. Leiner, C. Schörgenhofer, P. Kalhs, A. Schulenburg, C. Sillaber, M. Mitterbauer, W. R. Sperr, U. Jäger, K. Skrabs, H. Greinix, A. Hermann, W. Lamm

**Affiliations:** Bone Marrow Transplant Unit, Department of Medicine I, Medical University of Vienna, Vienna, Austria; Department of Medicine III, Division of Nephrology and Dialysis, Medical University of Vienna, Vienna, Austria; Department of Medicine I, Clinical Division of Hematology, Medical University of Vienna, Vienna, Austria; Division of Hematology, Medical University of Graz, Graz, Austria; Department of Medicine I, Intensive Care Unit 13i2, Medical University of Vienna, Vienna, Austria; Department of Medicine I, Clinical Division of Oncology, Medical University of Vienna, Waehringer Guertel 18-20, 1090 Vienna, Austria

**Keywords:** Hodgkin lymphoma, Allogeneic HSCT, Alemtuzumab-BEAM

## Abstract

**Purpose:**

Treatment of refractory Hodgkin disease deserves specific considerations. Recently, alemtuzumab-BEAM has been introduced in allogeneic hematopoietic stem cell transplantation (HSCT) in these patients.

**Methods:**

We retrospectively analyzed the outcome of 20 patients with relapsed/refractory Hodgkin’s lymphoma (HL) who received allogeneic HSCT following conditioning therapy with alemtuzumab-BEAM.

**Results:**

Treatment-related toxicity was tolerable. Half of the patients (50 %) had infections. Of these, 50 % were found to have pneumonia or catheter-related infections. In 20 %, an oral mucositis was observed. Acute graft-versus-host disease (GvHD) (≥grade 2) was seen in three patients. Complete remission (CR) could be achieved in 17 patients (85 %), 2 patients had persistent Hodgkin disease, and 1 patient died from infection prior to CR evaluation. Median progression-free survival and overall survival were 17.9 and 67.5 months, respectively. From the 17 CR patients, 8 had a relapse after a median of 10 months. Notably, of the eight patients relapsing after HSCT, all patients received another salvage treatment and four patients are still alive, whereas the other four patients died due to further progress. Six out of the remaining nine patients are still in CR, whereas the other three died from chronic GvHD and multi-organ failure. Overall, seven patients experienced chronic GvHD.

**Conclusion:**

In summary, alemtuzumab-BEAM is a well-tolerated conditioning therapy for allogeneic HSCT with high response rates in refractory HL.

## Introduction

The majority of patients with Hodgkin’s lymphoma show a response to conventional chemotherapy with response rates of 80 %. For patients with advanced Hodgkin’s disease, first-line therapy consists of chemotherapy with remission rates of up to approximately 80 % (Santoro et al. 1987). For patients with refractory/relapsed disease, the combination with high-dose chemotherapy followed by autologous hematopoietic stem cell transplantation (HSCT) is standard of therapy with remission rates of 50 % (Majhail et al. 2006; Schmitz et al. 2002). In case of relapse after autologous HSCT, various salvage regiments have been published. Brentuximab vedotin, nivolumab and bendamustine have shown promising results with overall response rates of 75, 87, and 53 %, respectively (Ansell et al. 2015; Moskowitz et al. 2013; Younes et al. 2012). In case of a related or unrelated donor, patients eligible for high-dose chemotherapy can also undergo allogeneic HSCT. However, a higher incidence of transplant-related mortality (TRM) and/or of graft-versus-host disease (GvHD) has to be considered (Milpied et al. 1996). To overcome these problems, reduced intensity conditioning regimens including BEAM-alemtuzumab have recently been developed.

The combination of alemtuzumab and BEAM as conditioning therapy before allogeneic HSCT showed promising results in patients with follicular lymphoma and lymphoproliferative disorders (Cull et al. 2000; Ingram et al. 2008). In this novel treatment scheme, alemtuzumab, a humanized monoclonal antibody to the panlymphoid antigen CD52, is combined with conventional chemotherapy with BEAM (carmustine, cytarabine, etoposide, and melphalan), a frequently used conditioning therapy in autologous HSCT (Mills et al. 1995).

The introduction of alemtuzumab was shown to result in a lower incidence of GvHD (7–17 %) compared with non-T cell-depleted regimens and has been investigated in patients with lymphoproliferative disorders and follicular lymphoma (Faulkner et al. 2004; Noriega et al. 2014).

In this retrospective, single-center analysis, we report on the outcome of 20 patients with relapsed/refractory HL undergoing allogeneic SCT with alemtuzumab-BEAM as conditioning therapy at our department.

## Patients and methods

### Patients

In this study, we retrospectively analyzed 20 patients with Hodgkin’s lymphoma who received allogeneic HSCT after conditioning therapy with alemtuzumab-BEAM at the Medical University of Vienna between 2004 and 2014. The median age at the time of allogeneic transplantation was 32 years (range 21–43 years). All patients had relapsed/refractory disease, and predominant stages were I and II (55 %), III (15 %), and IV (30 %) at time of primary diagnosis. A detailed list of prior chemotherapies and baseline characteristics is outlined in Table 1. Sites involved with HL immediately before allogeneic HSCT were lymph nodes in all patients (100 %). The most commonly used initial therapy consisted of ABVD (doxorubicin, bleomycin, vinblastine, and dacarbazine) (60 %) followed by BEACOPP (bleomycin, etoposide, cyclophosphamide, vincristine, procarbazine, and prednisone) (35 %). All but three patients (85 %) had received prior autologous HSCT. The median number of treatment lines prior to allogeneic HSCT was 5 (range 3–9). At the time of allogeneic HSCT, three patients (15 %) were in complete remission, seven (35 %) in a partial remission, and ten (50 %) had progressive disease. HSCT was performed with HLA-identical siblings (*n* = 10) or matched unrelated donors (URDs) (*n* = 10). All patients consented to treatment according to institutional guidelines and to anonymized assessments and analysis of data regarding outcome of therapy. The local ethical committee of the Medical University of Vienna approved all analyses.

**Table 1:** Baseline characteristics

Number of patients (*n* = 20)	Number (%)
Age median (range) in years at diagnosis	29 (19–41)
Age median (range) in years at allo ASCT	32 (21–43)
Gender	
Male	12 (60)
Female	8 (40)
Hodgkin lymphoma subtype	
Nodular sclerosis subtype	16 (80)
Lymphocyte-predominant subtype	4 (20)
Staging (at diagnosis)	
I + II	11 (55)
III	3 (15)
IV	6 (30)
B-symptoms at diagnosis	
Yes	9 (45)
No	11 (55)
Frontline therapy	
ABVD	12 (60)
BEACOPP	7 (35)
Cisplatine/gemcitabine	1 (5)
Prior autologous HSCT	
Yes	17 (85)
No	3 (15)
Prior therapy lines before allogeneic HSCT	
Median (range)	5 (3–9)
Current status	
Dead	10 (50)
Alive	10 (50)

ABVD, doxorubicin, bleomycin, vinblastine, and dacarbazine; BEACOPP, bleomycin, etoposide, doxorubicin, cyclophosphamide, vincristine, procarbazine, and prednisone

### Conditioning regimen

Conditioning therapy performed in all patients consisted of intravenous (i.v.) carmustine at 300 mg/kg body weight (BW) on day 6, cytarabine at 200 mg/kg BW on days 5 to 2, etoposide at 200 mg/kg BW on days 5 to 2, and melphalan at 140 mg/kg BW on day 1. Alemtuzumab at 1 mg absolute was given from days 5 to 1.

### GvHD prophylaxis and diagnosis

For GvHD prophylaxis, patients received cyclosporine A (CSA, target plasma level 150–200 ng/mL) and mycophenolate mofetil (15 mg/kg BW three times a day in patients with an unrelated donor and 15 mg/kg BW twice a day in patients with a related donor). In the absence of GvHD, CSA was tapered from day +100 and MMF treatment was stopped after full donor chimerism was established.

GvHD was clinically graded as 0 to IV for acute GvHD and mild, moderate, or severe for chronic GvHD according to the NIH guidelines (Filipovich et al. 2005; Sullivan et al. 1981; Thomas et al. 1975). All skin and gastrointestinal manifestations of acute GvHD were confirmed histologically based on appropriate biopsies. First-line therapy of acute GvHD consisted of corticosteroids at 2 mg/kg BW. For second-line therapy, extracorporeal photopheresis (ECP) was administered as previously reported (Greinix et al. 2000, 2010). Therapy of chronic GvHD consisted of corticosteroids and CSA as first-line therapy and ECP or basiliximab and etanercept as second-line therapy (Jagasia et al. 2015).

### Engraftment

In all patients, peripheral blood (PB) and reticulocyte counts were determined on a daily basis, starting 7 days before HSCT until hematopoietic engraftment. Absolute neutrophil counts (ANCs) were calculated from leukocyte and differential counts. Engraftment was defined as an ANC of at least 0.5 G/L for at least 3 days, untransfused platelet counts of at least 20 G/L, and independence from RBC transfusions. Chimerism analyses were performed serially on unseparated PB, CD3+, and CD33+ cells on days 28, 56, 84, and 180 and every 6 months thereafter.

### Supportive care

All patients received red cell concentrates to maintain a hemoglobin level >8.0 g/dl. Platelet transfusions were administered to keep platelet counts >20 G/L. All blood products were irradiated with 30 Gy. Approved informed consent was obtained from all patients. All patients were hospitalized in isolation rooms with laminar air flow or reverse isolation and received Pneumocystis carinii prophylaxis with cotrimoxazole and acyclovir for cytomegalovirus prophylaxis according to established guidelines (Winston and Gale 1991).

### Statistical analysis

Overall survival (OS) was calculated from the day of allogeneic HSCT until death from any cause. Patients who were alive or lost to follow-up were censored. Progression-free survival (PFS) was calculated from the day of allogeneic HSCT until relapse and/or death. Transplant-related mortality (TRM) was defined as mortality after allogeneic HSCT not related to relapse. The probabilities were estimated using the Kaplan–Meier method. The software package SPSS 17.0 (SPSS Inc. 1999) was used for these statistical analyses. Transplant-related mortality was defined as mortality after allogeneic HSCT not related to relapse.

## Results

### Hematologic toxicity and engraftment

Patients received a median number of 2 (range 0–16) red cell concentrates and a median of 2 (range 1–17) platelet transfusions, respectively. The median days to neutrophil and platelet engraftment for the remaining 18 patients were 15 (range 10–25) and 10 (range 10–35), respectively. In all but two patients, successful engraftment was documented. One out of these two patients subsequently received an autologous HSCT, the other one received umbilical cord blood transplantation, and both engrafted successfully thereafter.

### Outcome and survival

Median time from diagnosis to allogeneic HSCT was 30.7 months (range 12.8–172.8). Following allogeneic HSCT, 17 patients (85 %) achieved CR, 2 patients (10 %) had progressive disease, and one patient (5 %) died prior to CR evaluation. A complete chimerism was detected in 16 patients (75 %), whereas a mixed chimerism was detected in 3 patients (15 %) on day 28. Two out of them experienced full donor chimerism after tapering immunosuppression. The remaining patient died before chimerism analysis could be performed.

Eight out of the 17 CR patients showed a recurrence of HL after allogeneic HSCT after a median time of 10 months (range 3–20). Salvage therapy was administered in all patients. One patient received ibritumomab tiuxetan, three patients received cisplatin/gemcitabine/dexamethasone, one patient received brentuximab, and three patients received ICE (ifosfamide, carboplatin and etoposide). Four out of these eight patients are still alive, and four patients died due to further PD (Table 2).

**Table 2:** Transplantation data

	Number (%)
Response before allogeneic HSCT	
Complete remission	3 (15)
Partial remission	7 (35)
Progressive disease	10 (50)
Time from diagnosis to allogeneic ASCT	30.7 (12.8–172.8)
Conditioning	
Campath-BEAM	20 (100)
CD34+ cells transplanted	
Median (range)	6.4 (1.5–8.7)
Median (range) time to ANC >0.5 G/L in days	15 (10–25)
Median (range) time to PLT >20 G/L in days	10 (6–35)
Donor	
Sibling	10 (50)
Unrelated donor	10 (50)
Mismatch	
Yes (B-MM)	1 (5)
No	19 (95)
Chimerism state	
Complete chimerism	16 (75)
Mixed chimerism	3 (15)
Not available	2 (10)
Response to allogeneic ASCT	
Complete remission	17 (85)
Progressive disease	2 (10)
Not available	1 (5)
Progression-free survival (ASCT-PD) (months) (median (95 % CI)	17.9 (10.3–25.7)
Overall survival (ASCT-last follow-up/death)	67.5 (0–146.1)
First treatment after ASCT for relapsed disease (*n* = 8)	
Cisplatin/gemcitabine/dexamethasone	3 (38)
Ifosfamide/carboplatin/etoposide	3 (38)
Other	2 (24)
Treatment-related mortality	
Yes	2 (10)
No	18 (90)
Immunosuppressive therapy	
Cyclosporine A	5 (25)
Cyclosporine A/mycophenolate mofetil	14 (70)
Tacrolimus	1 (5)

BEAM, carmustine, cytarabine, etoposide, and melphalan

Six out of the nine patients who initially developed a CR are still in complete remission and alive, whereas the other three patients died due to septic multi-organ failure due to chronic GvHD without progression of their HL years after allogeneic HSCT.

**Fig. 1:**
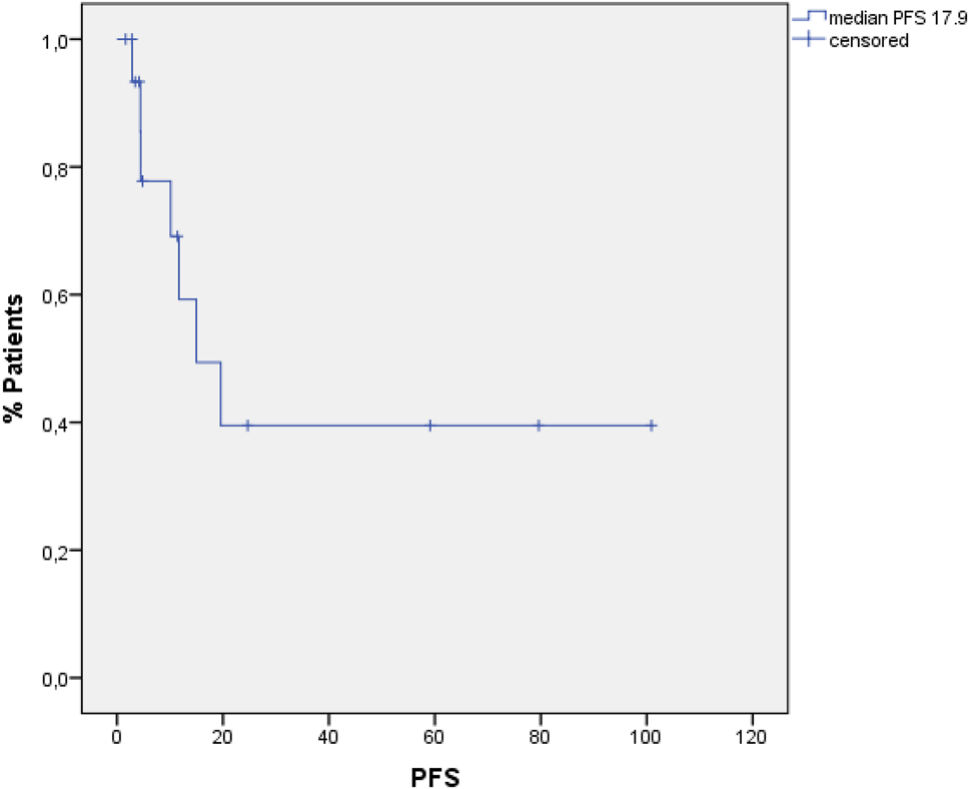
PFS for all patients

**Fig. 2:**
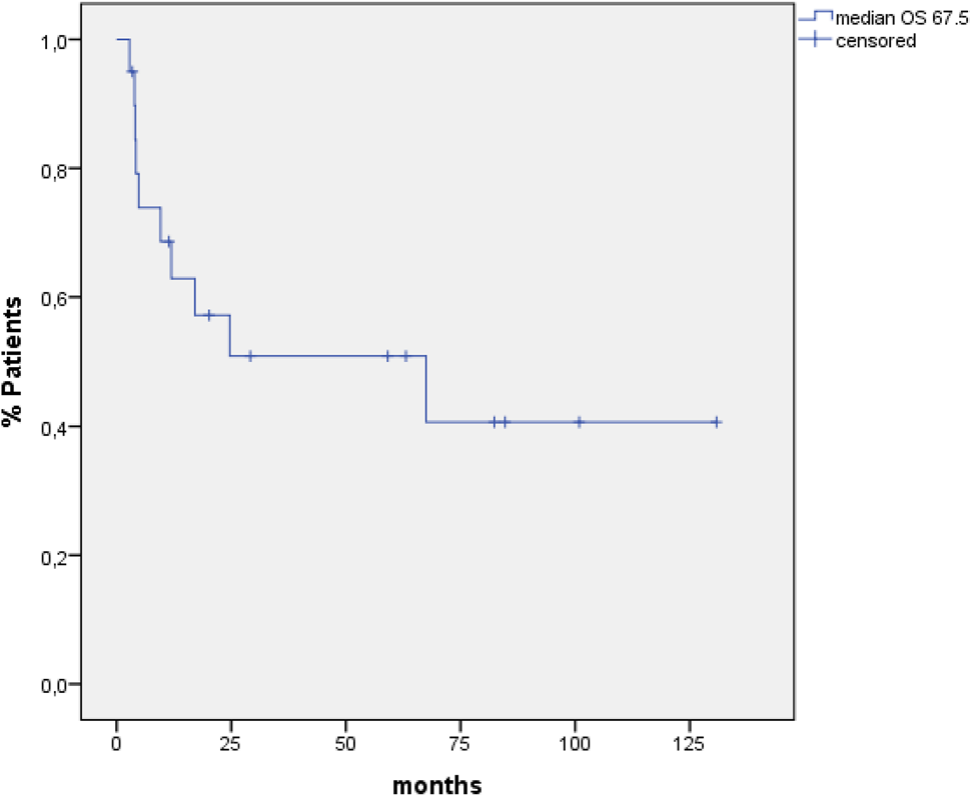
OS for all patients

Median PFS for all 20 patients was 17.9 months (95 % CI 10.3–25.7) (Fig. 1), and median OS was 67.5 months (95 % CI 0–146.1) when calculated from time point of allogeneic HSCT until death or last follow-up (Fig. 2).

### Regimen-related toxicity and GvHD

Table 3 represents regimen-related toxicity and GvHD. The conditioning therapy with alemtuzumab-BEAM showed a tolerable toxicity profile, which was well manageable. Side effects were only rated grade 1 and grade 2 and consisted of oral mucositis (20 %), diarrhea (20 %), and moderate infection (50 %). Fifty percent of infections were catheter related; thus, these catheters were removed and antimicrobial therapy was started subsequently, and the other five patients (50 %) developed pneumonia.

**Table 3:** Toxicity profile at peri-transplantation period and GvHD after stem cell transplantation

*N* = 20	Number (%)
Oral mucositis	4 (20)
Nausea	3 (15)
Diarrhea	4 (20)
Infection (*n* = 10)	10 (50)
Pneumonia	5 (50)
Catheter-related infection	5 (50)
Acute GvHD (≥grade 2)	
Intestine GvHD	1 (5)
Skin GvHD	2 (10)
Chronic GvHD (*n* = *7*)	
Mild	4 (58)
Moderate	1 (14)
Severe	2 (28)

Three out of 20 patients (29 %) developed acute GvHD ≥ grade 2 (intestine *n* = 1; skin *n* = 2). Seven (35 %) out of 20 patients developed chronic GvHD (mild *n* = 4; moderate *n* = 1; severe *n* = 2).

## Discussion

In this retrospective single-center analysis, we evaluated the efficacy of conditioning therapy with alemtuzumab-BEAM in 20 patients with relapsed/refractory HL. All patients had a median of 5 prior therapy lines (range 3–9). All but three patients had received an autologous HSCT due to progressive disease. Side effects of our conditioning therapy were rare and manageable and consisted mainly of oral mucositis and diarrhea. Median PFS and OS were 17.9 and 67.9 months, respectively. While the results of this small retrospective analysis have to be interpreted with caution, our data have a promising option for patients with refractory HL.

Allogeneic HSCT with myeloablative conditioning is associated with high TRM and discussed controversially in cases with classical HL (Sureda and Schmitz 2002). For patients with relapsed/refractory disease, however, it is the only possible curative therapy. In our study, patients with classical as well as with lymphocyte-predominant subtype responded to allogeneic HSCT. This is in line with previous reports showing a response rate of 62–71 % (Anderlini et al. 2008; Robinson et al. 2002; Sureda et al. 2012).

Different reduced intensity conditioning therapies have been reported (Anderlini et al. 2008; Peggs et al. 2005; Robinson et al. 2002). Anderlini et al. evaluated a treatment schedule with fludarabine–melphalan as conditioning therapy before allogeneic HSCT. Fifty-eight patients were included in this analysis; all but ten patients received prior autologous HSCT, and PFS and OS rates at 2 years were 20 and 48 %. This regimen was shown to be safe with a reduction in TRM (15 % at 2 years) (Anderlini et al. 2008). A similar TRM (2 patients) occurred in our patients.

Fludarabine–melphalan as conditioning therapy in 92 heavily pretreated patients was introduced in a phase II study (Anderlini et al. 2008). Fourteen patients died due to further progression after allogeneic HSCT, and 50 patients achieved a CR or PR. PFS and OS rates at 1 year were 47 and 71 % (Sureda et al. 2012). Robinson et al. reported different fludarabine-based regimens as preparative therapy for patients with different lymphomas. Fifty-two (28 %) patients had the diagnosis of HL. All included patients had a median of 3 prior therapy lines. PFS and OS rates at 2 years for these patients were 42 and 56 %, respectively. Relapse rate at 2 years after allogeneic HSCT was 45 % (Robinson et al. 2002). Because of the high relapse rate of >50 % after 1 year in the majority of reports, the addition of novel drugs in the conditioning protocols was of importance. Alemtuzumab-BEAM as conditioning therapy before allogeneic HSCT was first described in patients with lymphoproliferative disorders in the relapsed setting. This regimen was associated with a low incidence of acute GvHD (Cull et al. 2000). Another study in patients with relapsed/refractory lymphoproliferative disorders showed similar CR rates of approximately 73 % after allogeneic HSCT. Acute GvHD occurred in 17 % of all patients. Although only a few patients (5/65) with Hodgkin’s lymphoma were included in this study, the results are of interest, because the prevalence of GvHD was low again (Faulkner et al. 2004). In line with these reports, the rate of acute GvHD (15 %) was also low in our cohort and the response rate high (85 %).

The same conditioning therapy was applied in patients with follicular lymphomas (FL) (Ingram et al. 2008; Noriega et al. 2014). In the first study, the authors compared BEAM-autologous HSCT versus alemtuzumab-BEAM allogeneic HSCT. Looking at the results, substantial differences between the two treatment groups have to be considered. Likewise, more treatment lines prior to HSCT were reported in the group receiving alemtuzumab-BEAM. This together with the higher toxicity or the allogeneic regiment resulted in a higher prevalence of TRM and thus in a lower survival in the allogeneic arm. On the other hand, the relapse rate was significantly lower in the allogeneic arm (20 vs. 43 %; *p* = 0.01) (Ingram et al. 2008). The same treatment approach was used in two other studies (Faulkner et al. 2004; Noriega et al. 2014). Again a high rate of responses as well as a low incidence of GvHD was reported. However, the long-term PFS still remains low. Likewise, Truelove et al. (2015) show in their paper a PFS at 1 and 5 years of 48 and 36 %, respectively.

Similarly, in our study, the rate of early relapse after allogeneic HSCT was low. However, there was a markedly increased up to 52 % in the follow-up (10/19). Successfully engraftment was observed in 18 out of 20 patients. In our study, platelet count recovered 5 days earlier than granulocyte count.

Alemtuzumab-BEAM was a well-tolerated regimen with less side effects in our study. Infections were detected in ten patients with pneumonia and catheter-related infections 50 %, respectively. The high infection rate is most likely due to alemtuzumab. This is in accordance with other studies mentioned before (Cull et al. 2000; Faulkner et al. 2004).

Recently, new treatment strategies have been developed for patients with relapsed/refractory HL. In this regard, brentuximab, a novel CD30 antibody showed promising results with overall response rates (ORR) of approximately 75 %. The median PFS and OS were 5.6 and 20.5 months. Notably, 31 out of 102 patients remained in CR after 1.5 years. None of these patients underwent allogeneic HSCT (Younes et al. 2012). Another treatment option is bendamustine, which was shown to be an effective therapeutic approach in a phase II trial in patients with relapsed/refractory HL. The ORR was approximately 53 % with 12 patients qualifying for a CR (33 %). It is of particular interest that only patients who received bendamustine prior to autologous or allogeneic HSCT responded to this therapy (Moskowitz et al. 2013). Nivolumab, a PD-1-blocking antibody, proved efficacy in a cohort of 23 patients with relapsed/refractory HL, who had received an autologous HSCT and brentuximab. The ORR was 87 %, and the PFS rate at 24 weeks was 86 % (Ansell et al. 2015).

In the light of these novel strategies, it is of note that our patients were transplanted prior to the general availability of brentuximab and nivolumab still is an investigational drug. Thus, none of our patients had the possibility to receive one of these novel therapies.

Bendamustine was administered in two patients of our cohort who had a relapse after HSCT.

In summary, conditioning therapy with alemtuzumab-BEAM is a well-tolerated therapy with manageable toxicities. We observed a high CR rate (85 %) and a long PFS and OS with 17.9 and 67.5 months, respectively. Acute GvHD and chronic GvHD were rare and manageable. This combination is a feasible salvage treatment option in selected patients with relapsed/refractory HL after previous autologous HSCT.

## References

[CR1] Anderlini P, Saliba R, Acholonu S, Giralt SA, Andersson B, Ueno NT, Hosing C, Khouri IF, Couriel D, de Lima M, Qazilbash MH, Pro B, Romaguera J, Fayad L, Hagemeister F, Younes A, Munsell MF, Champlin RE (2008). Fludarabine-melphalan as a preparative regimen for reduced-intensity conditioning allogeneic stem cell transplantation in relapsed and refractory Hodgkin’s lymphoma: the updated M.D. Anderson Cancer Center experience. Haematologica.

[CR2] Ansell SM, Lesokhin AM, Borrello I, Halwani A, Scott EC, Gutierrez M, Schuster SJ, Millenson MM, Cattry D, Freeman GJ, Rodig SJ, Chapuy B, Ligon AH, Zhu L, Grosso JF, Kim SY, Timmerman JM, Shipp MA, Armand P (2015). PD-1 blockade with nivolumab in relapsed or refractory Hodgkin’s lymphoma. N Engl J Med.

[CR3] Cull GM, Haynes AP, Byrne JL, Carter GI, Miflin G, Rebello P, Hale G, Waldmann H, Russell NH (2000). Preliminary experience of allogeneic stem cell transplantation for lymphoproliferative disorders using BEAM-CAMPATH conditioning: an effective regimen with low procedure-related toxicity. Br J Haematol.

[CR4] Faulkner RD, Craddock C, Byrne JL, Mahendra P, Haynes AP, Prentice HG, Potter M, Pagliuca A, Ho A, Devereux S, McQuaker G, Mufti G, Yin JL, Russell NH (2004). BEAM-alemtuzumab reduced-intensity allogeneic stem cell transplantation for lymphoproliferative diseases: GVHD, toxicity, and survival in 65 patients. Blood.

[CR5] Filipovich AH, Weisdorf D, Pavletic S, Socie G, Wingard JR, Lee SJ, Martin P, Chien J, Przepiorka D, Couriel D, Cowen EW, Dinndorf P, Farrell A, Hartzman R, Henslee-Downey J, Jacobsohn D, McDonald G, Mittleman B, Rizzo JD, Robinson M, Schubert M, Schultz K, Shulman H, Turner M, Vogelsang G, Flowers ME (2005). National Institutes of Health Consensus Development Project on criteria for clinical trials in chronic graft-versus-host disease: I. Diagnosis and staging working group report. Biol Blood Marrow Transplant.

[CR6] Greinix HT, Volc-Platzer B, Kalhs P, Fischer G, Rosenmayr A, Keil F, Honigsmann H, Knobler RM (2000). Extracorporeal photochemotherapy in the treatment of severe steroid-refractory acute graft-versus-host disease: a pilot study. Blood.

[CR7] Greinix HT, Worel N, Knobler R (2010). Role of extracorporeal photopheresis (ECP) in treatment of steroid-refractory acute graft-versus-host disease. Biol Blood Marrow Transplant.

[CR8] Ingram W, Devereux S, Das-Gupta EP, Russell NH, Haynes AP, Byrne JL, Shaw BE, McMillan A, Gonzalez J, Ho A, Mufti GJ, Pagliuca A (2008). Outcome of BEAM-autologous and BEAM-alemtuzumab allogeneic transplantation in relapsed advanced stage follicular lymphoma. Br J Haematol.

[CR9] Jagasia MH, Greinix HT, Arora M, Williams KM, Wolff D, Cowen EW, Palmer J, Weisdorf D, Treister NS, Cheng GS, Kerr H, Stratton P, Duarte RF, McDonald GB, Inamoto Y, Vigorito A, Arai S, Datiles MB, Jacobsohn D, Heller T, Kitko CL, Mitchell SA, Martin PJ, Shulman H, Wu RS, Cutler CS, Vogelsang GB, Lee SJ, Pavletic SZ, Flowers ME (2015). National Institutes of Health Consensus Development Project on criteria for clinical trials in chronic graft-versus-host disease: I. The 2014 Diagnosis and staging working group report. Biol Blood Marrow Transplant.

[CR10] Majhail NS, Weisdorf DJ, Defor TE, Miller JS, McGlave PB, Slungaard A, Arora M, Ramsay NK, Orchard PJ, MacMillan ML, Burns LJ (2006). Long-term results of autologous stem cell transplantation for primary refractory or relapsed Hodgkin’s lymphoma. Biol Blood Marrow Transplant.

[CR11] Mills W, Chopra R, McMillan A, Pearce R, Linch DC, Goldstone AH (1995). BEAM chemotherapy and autologous bone marrow transplantation for patients with relapsed or refractory non-Hodgkin’s lymphoma. J Clin Oncol.

[CR12] Milpied N, Fielding AK, Pearce RM, Ernst P, Goldstone AH (1996). Allogeneic bone marrow transplant is not better than autologous transplant for patients with relapsed Hodgkin’s disease. European Group for Blood and Bone Marrow Transplantation. J Clin Oncol.

[CR13] Moskowitz AJ, Hamlin PA, Perales MA, Gerecitano J, Horwitz SM, Matasar MJ, Noy A, Palomba ML, Portlock CS, Straus DJ, Graustein T, Zelenetz AD, Moskowitz CH (2013). Phase II study of bendamustine in relapsed and refractory Hodgkin lymphoma. J Clin Oncol.

[CR14] Noriega V, Kaur H, Devereux S, Byrne J, Marcus R, Haynes A, Yallop D, McMillan A, Ingram W, Khan A, Kenyon M, Potter V, Russell N, Mufti GJ, Pagliuca A (2014). Long term follow-up of BEAM-autologous and BEAM-alemtuzumab allogeneic stem cell transplantation in relapsed advanced stage follicular lymphoma. Leuk Res.

[CR15] Peggs KS, Hunter A, Chopra R, Parker A, Mahendra P, Milligan D, Craddock C, Pettengell R, Dogan A, Thomson KJ, Morris EC, Hale G, Waldmann H, Goldstone AH, Linch DC, Mackinnon S (2005). Clinical evidence of a graft-versus-Hodgkin’s-lymphoma effect after reduced-intensity allogeneic transplantation. Lancet.

[CR16] Robinson SP, Goldstone AH, Mackinnon S, Carella A, Russell N, de Elvira CR, Taghipour G, Schmitz N, Lymphoma Working Party of the European Group for B, Bone Marrow T (2002). Chemoresistant or aggressive lymphoma predicts for a poor outcome following reduced-intensity allogeneic progenitor cell transplantation: an analysis from the Lymphoma Working Party of the European Group for Blood and Bone Marrow Transplantation. Blood.

[CR17] Santoro A, Bonadonna G, Valagussa P, Zucali R, Viviani S, Villani F, Pagnoni AM, Bonfante V, Musumeci R, Crippa F (1987). Long-term results of combined chemotherapy-radiotherapy approach in Hodgkin’s disease: superiority of ABVD plus radiotherapy versus MOPP plus radiotherapy. J Clin Oncol.

[CR18] Schmitz N, Pfistner B, Sextro M, Sieber M, Carella AM, Haenel M, Boissevain F, Zschaber R, Muller P, Kirchner H, Lohri A, Decker S, Koch B, Hasenclever D, Goldstone AH, Diehl V, German Hodgkin’s Lymphoma Study G, Lymphoma Working Party of the European Group for B, Marrow T (2002). Aggressive conventional chemotherapy compared with high-dose chemotherapy with autologous haemopoietic stem-cell transplantation for relapsed chemosensitive Hodgkin’s disease: a randomised trial. Lancet.

[CR19] Sullivan KM, Shulman HM, Storb R, Weiden PL, Witherspoon RP, McDonald GB, Schubert MM, Atkinson K, Thomas ED (1981). Chronic graft-versus-host disease in 52 patients: adverse natural course and successful treatment with combination immunosuppression. Blood.

[CR20] Sureda A, Schmitz N (2002). Role of allogeneic stem cell transplantation in relapsed or refractory Hodgkin’s disease. Ann Oncol.

[CR21] Sureda A, Canals C, Arranz R, Caballero D, Ribera JM, Brune M, Passweg J, Martino R, Valcarcel D, Besalduch J, Duarte R, Leon A, Pascual MJ, Garcia-Noblejas A, Lopez Corral L, Xicoy B, Sierra J, Schmitz N (2012). Allogeneic stem cell transplantation after reduced intensity conditioning in patients with relapsed or refractory Hodgkin’s lymphoma. Results of the HDR-ALLO study—a prospective clinical trial by the Grupo Espanol de Linfomas/Trasplante de Medula Osea (GEL/TAMO) and the Lymphoma Working Party of the European Group for Blood and Marrow Transplantation. Haematologica.

[CR22] Thomas E, Storb R, Clift RA, Fefer A, Johnson FL, Neiman PE, Lerner KG, Glucksberg H, Buckner CD (1975). Bone-marrow transplantation (first of two parts). N Engl J Med.

[CR23] Truelove E, Fox C, Robinson S, Pearce R, Perry J, Kirkland K, McQuaker G, Pagliuca A, Johnson P, Russell N, Cook G, British Society for B, Marrow T (2015). Carmustine, etoposide, cytarabine, and melphalan (BEAM)-campath allogeneic stem cell transplantation for aggressive non-hodgkin lymphoma: an analysis of outcomes from the British Society of Blood and Marrow Transplantation. Biol Blood Marrow Transplant.

[CR24] Winston DJ, Gale RP (1991). Prevention and treatment of cytomegalovirus infection and disease after bone marrow transplantation in the 1990s. Bone Marrow Transplant.

[CR25] Younes A, Gopal AK, Smith SE, Ansell SM, Rosenblatt JD, Savage KJ, Ramchandren R, Bartlett NL, Cheson BD, de Vos S, Forero-Torres A, Moskowitz CH, Connors JM, Engert A, Larsen EK, Kennedy DA, Sievers EL, Chen R (2012). Results of a pivotal phase II study of brentuximab vedotin for patients with relapsed or refractory Hodgkin’s lymphoma. J Clin Oncol.

